# The Correlation of Body Mass Index with Risk of Recurrence in Post-Menopausal Women with Breast Cancer Undergoing Fluorodeoxyglucose Positron Emission Tomography/Computed Tomography

**DOI:** 10.3390/jcm13061575

**Published:** 2024-03-09

**Authors:** Natale Quartuccio, Salvatore Ialuna, Sabina Pulizzi, Dante D’Oppido, Mauro Antoni, Antonino Maria Moreci

**Affiliations:** Nuclear Medicine Unit, Ospedali Riuniti Villa Sofia-Cervello, 90146 Palermo, Italy; s.ialuna@villasofia.it (S.I.); medicinanucleare@villasofia.it (A.M.M.)

**Keywords:** PET/CT, breast cancer, body mass index

## Abstract

**Background:** The aim of this study was to investigate whether high body mass index (BMI) increases the risk of recurrence and correlates with higher glucose uptake in recurrent lesions in post-menopausal female patients with breast cancer. **Methods:** A hospital database was searched to retrieve breast cancer patients who had undergone an [^18^F]FDG PET/CT scan before neoadjuvant chemotherapy and curative-intent surgery. BMI was calculated at the baseline [^18^F]FDG PET/CT scan. There was a median follow-up of 5 years after the baseline PET/CT scan to identify recurrence in the breast (T_rec); lymph nodes (N_rec); and distant locations (M_rec). Furthermore, SUVmax was measured at the sites of recurrence. A chi-square test was used to investigate any difference in the frequency of any recurrence, T_rec, N_rec, and M_rec, between overweight women (BMI ≥ 25 kg/m^2^) and women with a BMI < 25 kg/m^2^ (*p* < 0.05). SUVmax was compared using a *t*-test (*p* < 0.05) between the two groups. **Results:** A total of 142 post-menopausal patients (BMI: 26.84 ± 5.59; 84 overweight and 58 with normal weight) were retrieved from the database. There were 48 recurrences at the follow-up. The chi-square test demonstrated in overweight women an increased frequency of any recurrence (35 vs. 13; *p* = 0.025) and T_rec (15 vs. 2; *p* = 0.018) and a higher T_rec SUVmax (4.74 ± 2.90 vs. 1.85 ± 0.63; *p* = 0.09) compared to women with a BMI < 25 kg/m^2^. **Conclusions:** BMI seems to correlate with an increased rate of recurrence, especially in the breast, and a higher glucose uptake in post-menopausal patients with recurrent breast cancer.

## 1. Introduction

Breast cancer (BC) is the most common type of cancer in the world in women and also the leading cause of death from cancer for them [1]. According to the International Agency for Research on Cancer, in 2020, between 675,493 and 694,633 women died from breast cancer worldwide, with an age-adjusted rate of 13.6 per 100,000 [2]. By 2040, it is anticipated that due solely to population growth and an aging populace, the toll of breast cancer could soar to over 3 million new cases annually, resulting in 1 million deaths each year [3].

There are several environmental, genetic, and lifestyle risk factors. These include older age, genetic mutations to certain genes (such as BRCA1 and BRCA2), reproductive history, dense breasts, family history of BC, previous radiation therapy, exposure to certain drugs (e.g., diethylstilbestrol), smoking, alcohol intake, exposure to chemicals, hormone replacement therapy, lack of physical activity, and obesity. For the latter two risk factors, changes in life style are frequently advocated [4,5]. Although a number of observational studies have suggested that exercise in conjunction with weight loss may improve outcomes for patients with breast cancer, more studies are needed to determine whether weight loss might actually improve patient outcomes [6]. 

Indeed, the incidence of people who are overweight and obesity is increasing worldwide [7], and these conditions are considered risk factors for many types of cancers, possibly through modifications in metabolic activity and the immune status [8,9]. Although not completely exhaustive, body mass index (BMI) is a commonly used anthropometric parameter that relates body mass and the square of a person’s height [10]. BMI is commonly used to screen for and diagnose a person as being overweight (BMI 25–29.9 kg/m^2^) and obesity (BMI ≥ 30 kg/m^2^), categorizing obesity severity into different classes [11]. 

It has been observed that increased BMI may be a detrimental factor for the outcome of patients with breast cancer [12]. In a large cohort study, a total of 2148 patients, including 592 premenopausal and 1556 postmenopausal individuals, were divided into subgroups based on BMI distribution. A higher BMI was significantly associated with larger tumor sizes in both premenopausal (*p* = 0.01) and postmenopausal women (*p* < 0.001) [13]. In another study, 636 women were tracked for a median period of more than 13 years. The authors found that higher BMI generally indicates a quicker recurrence of the disease and reduced survival rates. However, this relationship appeared to be more pronounced among younger women, those with progesterone receptor-negative disease, and those with a higher number of affected lymph nodes [12]. Interestingly, a meta-analysis gathered data from 12 prospective cohort studies involving 22,728,674 participants and its findings suggested a slight positive association—indicating a 2% rise in breast cancer risk with every 5 kg/m^2^ increase in BMI. Surprisingly, for premenopausal women, a higher BMI seemed to lower breast cancer risk [14]. 

Early-stage breast cancer, confined to the breast or spread to nearby lymph nodes, is considered treatable with a success rate of 70–80%, thanks to advancements in treatment options. In contrast, advanced-stage breast cancer is not curable, but treatment aims to extend survival, manage symptoms, and enhance quality of life. Accurate staging of breast cancer is essential for guiding treatment decisions and clinical management [15]. Imaging is crucial in assessing patients with breast cancer. These individuals typically undergo various imaging examinations—like Ultrasound, Magnetic Resonance Imaging, Computed Tomography, lymphoscintigraphy to locate the sentinel lymph node, bone scans, and Positron Emission Tomography (PET)/Computed Tomography (CT) tailored to their cancer type and risk level. Additionally, the cancer’s attributes and clinical factors guide the selection of the most suitable treatment for these specific groups of patients [16]. While PET/CT is not currently a routine exam for early-stage breast cancer staging, it holds significance in evaluating systemic staging [17]. The most widely used PET radiopharmaceutical in oncology is fluorodeoxyglucose ([^18^F]FDG), which serves as a metabolic biomarker to assess glycolytic activity in tumors. [^18^F]FDG PET/CT is a well-established examination in the diagnostic workflow of BC both at staging and restaging [16]. Leitner et al. also found that greater tumor glucose uptake is generally observed in patients with BC with increased BMI compared to patients with normal BMI [18]. Increased uptake of [^18^F]F-FDG is generally associated with a worse outcome for patients, particularly in those with grade 3 tumors compared to those with grade 1 or 2 tumors [19]; nevertheless, the combined influence of being overweight may be even more detrimental for prognosis [4,14]. Indeed, a study explored the link between visceral adipose tissue (VAT) metabolic activity, assessed via preoperative [^18^F]FDG PET/CT scans, and axillary lymph node (ALN) metastasis in 173 postmenopausal luminal breast cancer patients. The results indicated that higher VAT metabolic activity, measured as the maximum standardized uptake value (SUVmax) ratio of VAT to subcutaneous adipose tissue (V/S ratio), was notably linked to ALN metastasis in luminal breast cancer. Patients with ALN metastasis demonstrated significantly higher V/S ratios compared to those without. Additionally, erythrocyte sedimentation rate, reflecting systemic inflammation, was notably higher in the ALN metastasis group among luminal BC patients. It exhibited a significant positive correlation with the V/S ratio, suggesting a potential association between VAT metabolic activity, systemic inflammation, and tumor aggressiveness in postmenopausal luminal breast cancer [9].

The aim of this article is to investigate whether a high body mass index (BMI) increases the risk of recurrence and correlates with higher glucose uptake in recurrent lesions in post-menopausal women with BC.

## 2. Materials and Methods

### 2.1. Patients

The hospital RIS/PACS system (Elephant v.2, Agfa HealthCare, Mortsel, Belgium) was searched by an author to retrieve post-menopausal women with breast cancer who had undergone a pre-operative [^18^F]FDG PET/CT scan before neoadjuvant chemotherapy and curative-intent surgery. Menopausal status was confirmed by means of a written declaration at the time of the [^18^F]FDG PET/CT scan by each patient involved in the study. 

Inclusion criteria comprised (a) histological confirmation of unilateral breast cancer; (b) availability of a pre-operative [^18^F]FDG PET/CT scan; (c) neoadjuvant and curative-intent surgery; (d) confirmation of post-menopausal state; (e) availability of clinical follow-up information in the hospital database. To ensure that premature menopause (before the age of 40 years) or early menopause (between the ages of 40 and 45 years) were not excluded, no age restrictions were applied to adult women in the database search. Exclusion criteria included execution of neoadjuvant radiotherapy. 

The interval between [^18^F]FDG PET/CT scan and surgery was 1–2 weeks. At the time of the baseline [^18^F]FDG PET/CT scan, BMI was calculated using the following formula: weight in Kg/(height in m)^2^. Patients were divided in two groups (high BMI and normal BMI) using a threshold of BMI ≥ 25 kg/m^2^ according to the recommendation of the World Health Organization [20]. 

Data on patient follow-up and recurrence were gathered from both medical records and the institutional tumor registry. Each patient received at least one PET/CT scan for follow-up purposes every year for a follow-up of 5 years after the baseline PET/CT scan. The follow-up was planned to identify recurrence in the breast (T_rec); lymph nodes (N_rec); distant locations (M_rec). Follow-up information included imaging scans (US, mammography, CT, MRI and PET/CT scans) and clinical consultations. Patients underwent clinical follow-ups every 6 to 12 months post-surgery.

Due to the retrospective design of the study, the local ethical committee granted a full waiver for ethical approval.

### 2.2. PET/CT Scans and Image Reconstruction

Before the PET/CT scan, all patients had to fast for at least six hours. Individuals whose fasting glucose levels exceeded 190 mg/dl were delayed until an appropriate treatment plan was arranged. After receiving a manual injection of [^18^F]FDG (3.7 MBq/Kg), the patients sat comfortably in a chair and received adequate hydration using an intravenous drip. The PET/CT scan was performed 60 ± 10 min later, using a standard imaging protocol that followed the guidelines of the European Association of Nuclear Medicine [21]. 

All the PET/CT scans were performed on a Discovery STE scanner (GE Healthcare, Milwaukee, WI, USA) equipped with a 16-slices CT scan. First, a low-dose CT scan (80 mA/s and 120 kV) for the attenuation correction of the PET emission data was carried out without the use of any contrast agent. Then, emission images, ranging from the base of the skull to mid-thigh, were acquired approximately for 3 min per bed position. If the BMI was high, the scanning duration was modified based on the BMI, extending by half a minute per frame. The PET images were reconstructed by an expert nuclear medicine physician in the axial, coronal, and sagittal planes using an ordered-subset expectation maximization (OSEM) algorithm, which involved 4 iterations with 16 subsets. The reconstructions were performed on a 128 × 128 matrix with a pixel size of 4.75 mm and a slice thickness of 2 mm. A Gaussian filter was applied to smooth out the images. An MIP image was also stored for each patient.

### 2.3. Imaging Analysis

Two blinded experienced nuclear medicine physicians (with more than 5 years of experience) reviewed serial PET/CT scans for each patient on a Xeleris workstation (GE Healthcare, Milwaukee, WI, USA). Each imaging scan was carefully examined to detect T_rec, N_rec, and M_rec over time. For each organ of recurrence, SUVmax, normalized to patient body weight, was measured by manually drawing a volume of interest (VOI) in the most [^18^F]FDG-avid lesion at visual analysis and then was recorded in a spreadsheet. If there was a disagreement between the two nuclear medicine physicians regarding the sites of recurrence, a consensus was ultimately achieved by consulting a further blinded nuclear medicine physician.

### 2.4. Statistical Analysis 

Categorical variables were outlined by their frequencies, while continuous variables were represented by their mean along with standard deviation and range. A chi-square test was used to investigate any difference in the categorical variables and in the frequency of any recurrence, T_rec, N_rec, and M_rec between overweight women (BMI ≥ 25 kg/m^2^) and women with a BMI < 25 kg/m^2^ (*p* < 0.05). SUVmax was compared between the patient groups for any recurrence, T_rec, N_rec, and M_rec using a two-sides t-test (*p* < 0.05). The gold standard to confirm the metastatic nature of the [^18^F]FDG-avid findings at the 5-year follow-up was based on pathology or, if not available, on a composite reference standard comprising all available imaging procedures. The statistical analysis was conducted using version 19.1.3 of MedCalc Statistical Software (developed by MedCalc Software based in Ostend, Belgium, available at https://www.medcalc.org, accessed on 7 January 2023).

## 3. Results

After performing the hospital database search and applying the inclusion criteria, 142 post-menopausal women (mean BMI: 26.84 ± 5.59, range: 17–44 kg/m^2^; mean age: 63.21 ± 11.02, range: 35–86 years) were found. The demographical information for the overweight (n = 84) and normal weight (n = 58) women is summarized in Table 1. 

**Figure 1:**
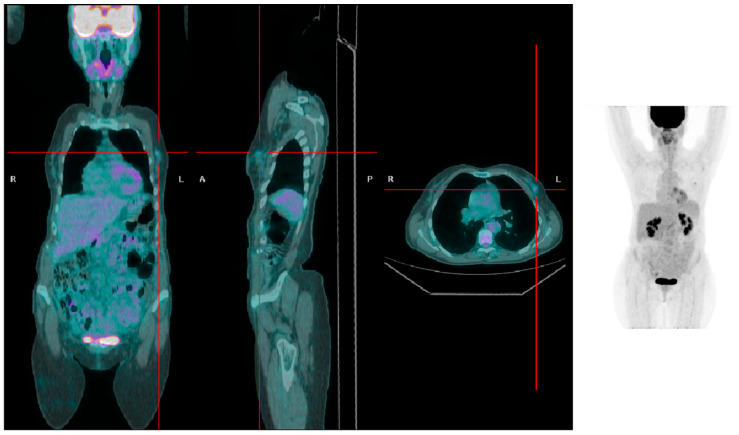
A patient with low BMI presenting with a left-sided breast tumor with mild [^18^F]FDG uptake (SUVmax 1.5).

**Figure 2:**
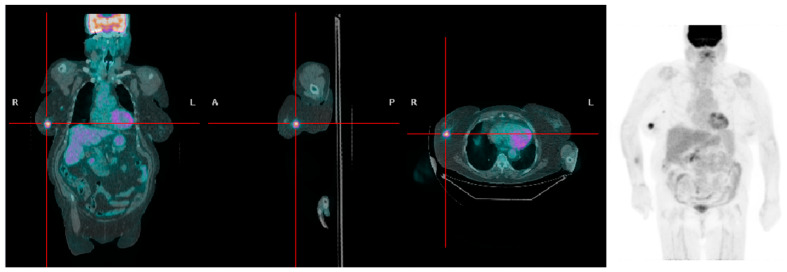
A patient with high BMI presenting with a right-sided breast tumor with high [^18^F]FDG uptake (SUVmax 6.3).

There were 48 recurrences at the follow-up (35 in the overweight group and 13 in patients with normal weight). The chi-square test demonstrated a higher frequency of recurrences in overweight women compared to women with a BMI < 25 kg/m^2^ (35 vs. 13; *p* = 0.025). The local and regional recurrence rates were 17.85% and 25% in the overweight patient group and 3% and 12% in the normal weight patient group, respectively. A significantly higher frequency of T_rec (15 vs. 2; *p* = 0.018) and a higher T_rec SUVmax (4.74 ± 2.90 vs. 1.85 ± 0.63, respectively; *p* = 0.09; Figure 1 and Figure 2) were found in overweight women compared to women with a BMI < 25 kg/m^2^. No statistically significant difference between the two groups was found for the frequency of N_rec and M_rec. SUVmax of N_rec was significantly higher in overweight patients compared to patients with BMI < 25 kg/m^2^ (6.57 ± 4.08 vs. 3.14 ± 1.62, respectively; *p* = 0.004).

## 4. Discussion

Over the past few decades, there has been an unprecedented surge in obesity, revealing increasingly apparent health repercussions. In 2014, globally, more than 1.9 billion adults were overweight (with a body mass index [BMI] of 25–29.9 kg/m^2^), and among them, over 600 million were classified as obese (BMI ≥ 30 kg/m^2^). While the link between obesity and the risk of diabetes and coronary artery disease is widely recognized, the full extent of obesity’s impact on cancer occurrence, morbidity, and mortality is not fully grasped. Obesity is correlated with a heightened likelihood of developing breast cancer, especially in postmenopausal women, and leads to more adverse outcomes for women across all age groups [6]. 

One primary concern in managing breast cancer therapy involves the ability to categorize patients who face a notable risk of disease progression. Obesity and being overweight pose a significant public health challenge. Studies, systematic reviews, and meta-analyses have consistently linked obesity to increased breast cancer risk [22]. Recent reports, such as the 2012 Annual Report to the Nation on Cancer, suggest that overweight and obese women have 1.13 and 1.25 times increased risks, respectively, of postmenopausal breast cancer compared to women of a normal weight [23]. Notably, a study focused on evaluating the impact of pre-diagnosis dietary habits and various patient factors associated with breast cancer incidence on its recurrence and post-treatment survival. Following 149 patients diagnosed with primary breast cancer between 1989 and 1991 for at least five years, the key findings were that higher intake of total energy and fats (especially saturated and monounsaturated fats) is linked to increased recurrence risk [24].

Excess body fat triggers various changes in the body that contribute to cancer development. Indeed, there is increased production of growth factors, like insulin and insulin-like growth factor 1 (IGF-1), which promote cell growth and division and higher levels of sex hormones, particularly estrogen, which can influence cancer risk. Furthermore, alterations in adipokines, such as visfatin, adiponectin, and leptin, play a role in immune regulation and tumor control. In addition, chronic inflammation and elevated levels of inflammatory cytokines promote oxidative stress, damaging cells and increasing cancer risk [4]. 

Although there are a few ways to gauge one’s level of fatness (waist circumference, waist-to-hip ratio, total body fat, and % body fat), BMI is the most often used method. A high BMI in BC patients at diagnosis has been linked to a higher chance of disease recurrence, development of BC metastases, and increased risk of death, particularly in post-menopausal women [4,14]. Other factors, such as the severity of the disease and receptor status, might influence this association. Ecker et al. explored the link between obesity and breast cancer recurrence using a mouse model. They found that high-fat diet-fed mice, classified as obese, had faster tumor recurrence compared to lean mice or those on a low-fat diet. The obese mice also showed physiological changes akin to obese individuals, like higher body fat percentage, glucose-related issues, and altered levels of certain biomarkers. Importantly, obese mice harbored more residual tumor cells after treatment, suggesting a potential causal relationship between obesity and the survival of these cells, indicating a heightened risk of breast cancer recurrence in obese individuals [25]. 

A limit of this study was its single-center and long-term retrospective design. Another limit of the study derives from the lack of additional exams in the definition of what is overweight. Indeed, BMI does not directly measure fat since weight comprises lean mass, bone, and fluid in addition to fat. BMI is useful for screening purposes but a physical exam is needed to confirm excess fat. BMI also does not consistently indicate how fat impacts health at specific BMI levels [11]. One significant constraint was the absence of histological evidence for all lesions, which was due to ethical and practical considerations. Further, we did not collect data on progression-free survival (PFS) and cancer-specific survival (CSS); we aim to examine the relationship between BMI and survival data in future studies. Another limit is the lack of estimation of muscular mass. Indeed, muscular tissue also contributes to BMI and a moderate increase in BMI may be associated with longer survival and better response to therapy in oncological patients according to some authors [26]. This intriguing observation is known as the “obesity paradox”. However, this beneficial effect diminishes as BMI levels reach morbid obesity. Elevated adipose tissue levels may indicate energy reserves that might prolong survival through the severe side effects of chemotherapy. These fat reserves could act as a reserve, which could increase survival time. The main source of estrogen in premenopausal women is the ovaries. However, in postmenopausal women, the synthesis of estrogen is mostly dependent on adipose tissue. Studies show that obese postmenopausal women have a higher chance of developing estrogen-dependent breast cancer. Increased inflammatory cells and biochemical markers are indicators of a systemic chronic inflammatory state that is brought on by obesity, which may have a connection to triple-negative breast tumors (TNBCs). There is no evidence connecting fat-induced inflammation to cancer, despite the well-established links between obesity, cardiovascular disease, and type 2 diabetes. Thus, it is imperative to elucidate if obesity-induced inflammation contributes significantly to the development of cancer and the underlying processes involved [4].

In the literature, the correlation between risk of recurrence of BC and increased BMI has been explored by only two clinical studies using [^18^F]FDG PET imaging. Lee et al. assessed the distribution of adipose tissue in the belly and gluteofemoral areas and recurrence-free survival (RFS) in patients with BC. The researchers assessed abdominal subcutaneous adipose tissue (SAT), visceral adipose tissue (VAT), and gluteofemoral adipose tissue by analyzing imaging data from 336 women. They discovered correlations between RFS and the abdomen-to-gluteofemoral adipose tissue volume ratio (AG volume ratio). A higher risk of recurrence was connected with increasing abdominal SAT volume and AG volume ratio, but a reduced risk was linked to increased gluteofemoral adipose tissue volume. Even after accounting for clinical/histological variables, these correlations were still significant [27]. Hyun et al. examined the prognosis of 332 patients with BC in relation to the combined effects of BMI and SUV, which were assessed in pretreatment [^18^F]FDG PET/CT, followed by curative resection. The authors discovered that obesity and having a large tumor SUV were independent risk factors for the recurrence of the disease. Positive status for hormone receptors was linked to a successful outcome. Once clinical stage and tumor subtype were taken into account, patients who were overweight and had a high tumor SUV had a twofold increased risk of recurrence compared to those who were normal weight or had a low SUV. Therefore, regardless of clinical stage, the combination of tumor SUV data and BMI status enhanced breast cancer risk classification [28].

Beyond VAT, brown adipose tissue (BAT) may also deserve further attention in future studies. Pace et al. looked into the relationship between genetic tumor characteristics and brown adipose tissue (BAT) activation in breast cancer. Out of 79 patients diagnosed with ductal breast cancer, 15.2% had activated BAT. BAT-activated patients were less likely to metastasize, had lower body mass indices (BMIs), and were younger. Hormone receptors, Ki67, grade, or molecular subtypes did not change significantly. A significant difference in BAT activation was not observed among patients under 55 years old with a BMI less than 26. Total metabolic activity (TMA) and BMI were observed to significantly correlate inversely in patients with BAT activation. In contrast to grade 3 patients, grade 2 patients had greater TMA and SUVmax. There were no discernible variations in TMA and SUVmax between patients who had lymph node metastases and those who did not. Among molecular types, there were notable variations in SUVmax and TMA, with luminal B patients exhibiting greater values. According to the study, there may be a connection between BAT activation and favorable prognostic elements for breast cancer, namely luminal B cancer type and intermediate tumor grade [29].

In light of the recent literature, another limit of our study is the analog nature of the PET/CT scanner used. Indeed, an improved image quality with digital scanners has been demonstrated when compared with analogue scanners, especially in overweight and obese subjects [30], alongside more accurate quantification of glucose uptake, a meaningful increased number of metastases, and superior small-lesion detection [31]. The intensity of the SUV in the tumor has been utilized as a substitute measure for the time it takes for BC to progress; it is conceivable that volume-based measures may also show a link with BMI and mirror different risks of recurrence. The impact of health and treatment status could not be examined here. Tumor growth in obese people may be impacted by chronic hyperglycemia, increasing circulating insulin levels, elevated glucocorticoids, increased inflammation, and other associated processes on systemic glucose metabolism. Another additional development for future studies could be the inclusion of volume-based parameters. 

BC typically exhibits increased expression of GLUT 1–3, making it detectable through [^18^F]FDG PET/CT scans. [^18^F]FDG PET/CT is a single valuable imaging technique that efficiently depicts metastatic illness at recurrence. Our findings, in keeping with the findings of Leitner et al. [18], confirm that increased body fat may contribute further to increased [^18^F]FDG accumulation. Increased BMI is a detrimental factor in many other types of cancer. Yugawa et al. observed a statistically significant correlation between BMI and SUVmax in patients with cholangiocarcinoma [8]. Likewise, in another study, increased BMI was found to correlate with poor prognosis and an increased risk of recurrence among patients undergoing curative resection for intrahepatic cholangiocarcinoma [32]. The correlation between obesity and risk of lymph node metastastasis in endometrial cancer was instead the aim of the study of Pahk et al. In particular, in their study, visceral adipose tissue (VAT) inflammation was measured by comparing the radioactivity of VAT to the radioactivity of subcutaneous adipose tissue (SAT). The group of patients with lymph node (LN) metastasis had significantly higher VAT inflammation than the group of patients without LN metastasis [33]. In another study of 59 patients who had chronic hepatitis C and underwent surgery for liver cancer after successful antiviral treatment, the researchers found that obesity and non-anatomical resection were key factors linked to the return of cancer after surgery. Obese patients had almost three times the risk of cancer recurrence compared to non-obese patients. Therefore, the study suggests that dealing with obesity could be vital in improving outcomes for these patients [34]. 

Recognition of being overweight or obesity as additional negative prognostic factors, along with [^18^F]FDG uptake, may prompt additional surveillance of patients and suggest more aggressive therapeutic strategies. In addition, for overweight or obese women with BC, a modest weight decrease may be advised. Further ad hoc design studies are warranted to support these assumptions. 

## 5. Conclusions

BMI seems to correlate with an increased rate of recurrence, especially in the breast, and a higher glucose uptake in post-menopausal patients with recurrent breast cancer. More extensive research samples are required to validate these preliminary results and their possible implementation in therapeutic settings.

## Figures and Tables

**Table 1:** Demographical information of overweight and normal weight patients. D: ductal, L: lobular.

	Overweight	Normal Weight	*p*.
n.	84	58	
BMI	31.43 ± 5.59	22.91 ± 2.55	<0.001
Age	66.01 ± 11.11	60.5 ± 11.45	0.005
Histotype	D: 76; L: 8	D: 53; L: 5	ns
Stage	T1: 60, T2: 24	T1: 43; T2: 15	ns
Any recurrence	35	13	0.45
T_rec	15	2	0.018
SUVmax T_rec	4.74 ± 2.9	1.85 ± 0.63	0.09
N_rec	21	7	0.18
SUVmax N_rec	6.57 ± 4.08	3.14 ± 1.62	0.004
M_rec	26	10	0.27
SUVmax M_rec	5.94 ± 4.45	5.71 ± 4.58	0.15

## Data Availability

Data can be provided upon reasonable request.
